# Is the gemcitabin-cisplatin combination the optimal induction chemotherapy for non-Asian patients with nasopharyngeal carcinoma (NPC)? Insights from a cohort in northeastern Morocco

**DOI:** 10.3332/ecancer.2025.1829

**Published:** 2025-01-22

**Authors:** Oumaima Talbi, Khadija Hinaje, Samia Mhirech, Kaoutar Maadin, Imad Chakri, Lamiae Amaadour, Karima Oualla, Zineb Benbrahim, Touria Bouhafa, Nawfel Mellas, Samia Arifi

**Affiliations:** 1 Department of Medical Oncology, Hassan II University Hospital, Fez 30000, Morocco; 2 Department of Radiotherapy, Chu Hassan II of Fes, Fez 30000, Morocco; 3 Laboratory of Epidemiology and Research in Health Sciences, Faculty of Medicine and Pharmacy, Sidi Mohamed Ben Abdallah University, Fes, Morocco

**Keywords:** nasopharyngeal carcinoma, induction chemotherapy, radiotherapy

## Abstract

According to the latest guidelines for nasopharyngeal carcinoma (NPC), induction chemotherapy (IC) followed by concomitant chemoradiation therapy is recommended as the preferred standard of care for patients with locally advanced NPC (stage III-IVA). However, the optimal regimen for IC in patients with locally advanced NPC remains uncertain.

**Purpose:**

We conducted a retrospective study to compare the effectiveness and tolerability of two platinum-based IC regimens; gemcitabine – Cisplatin (GC), and doxorubicin-Cisplatin (DP) in the treatment of newly diagnosed locally advanced NPC. The main objective of this study was to compare efficacy, including objective response rates (ORRs), progression-free survival (PFS), overall survival (OS) and safety.

**Results:**

105 patients were satisfied with the eligibility criteria and were, therefore, selected for analysis (62 patients in the DP group and 43 in the GC group), including 65 men and 40 women, with a mean age of 49.5 years (range = 19–79 years) and a Karnofsky score ranging from 87% to 100%. 34% of patients were diagnosed at stage IVA.

In the DP group, 3% of patients (2 out of 62) achieved a complete response complete response (CR), 60% achieved a partial response (PR), 25% remained stable S and 19% experienced progression. In the GC group, 2% of patients (1 out of 43) achieved a CR, 39.5% achieved a PR, 39.5% remained stable and 19% experienced progression. A statistically significant difference in PR was observed between the two groups (*p* = 0.028), and the difference in terms of progression is approaching the limit of significance (*p* = 0,06) after a median follow-up of 27 months (5.3–82). The 2-year PFS was 70% in the DP group compared to 80% in the GC group; the 2-year OS was 75% in the DP group and 90% in the GC group. No significant survival difference was observed between the two groups.

Patients in the DP group exhibited less grade 3–4 thrombocytopenia but more grade 3–4 leukopenia and neutropenia compared to the GC group.

**Conclusion:**

In patients with locally advanced NPC, DP-based IC demonstrated superior ORR compared with the GC regimen, with acceptable toxicity. Further studies are required to validate these results.

## Introduction

Nasopharyngeal carcinoma (NPC) is a head and neck cancer characterised by an imbalanced global geographic distribution, with the highest incidence in the regions of Southern China, Southeast Asia and North Africa [1]. NPC exhibits a notable gender disparity, with a higher risk in males [2]. Furthermore, almost 70% of patients are diagnosed with locally advanced stage II–IVB disease [3]. The most common histological type of NPC in Maghreb countries is undifferentiated nasopharyngeal carcinoma (UCNT) [4]. Morocco is an endemic area for UCNT, with an incidence considered intermediate by the World Health Organisation [5], with a rate of 3.7 cases per 100,000 according to the Casablanca Cancer Registry [5]. Given the anatomically challenging location for surgical intervention and the radiosensitivity of NPC, radiotherapy constitutes the primary treatment approach. However, its effectiveness in controlling locally advanced NPC is somewhat limited. As a result, previous studies have established concomitant chemoradiation therapy (CCRT) as the standard of care for locally advanced NPC, specifically in stages III and IVA [6].

There is a growing bodof evidenceto support the use of platinum-based induction chemotherapy (IC) followed by CCRT for the treatment of locally advanced NPC. Several trials have shown a survival advantage compared to CCRT alone [7]. Nevertheless, the ideal regimen for IC remains uncertain.

In our local practice, IC has consistently been the standard of care for stage III and IVA NPC, with a preference for a regimen consisting of 5FU-cisplatin (PF) or doxorubicin and cisplatin (DP) administered every 3 weeks for 3 cycles. However, following the publication of the phase III randomised controlled trial in 2019 that showed a survival benefit for gemcitabine-cisplatin (GC) IC followed by CCRT, and the subsequent update of the international guidelines recommending GC as the preferred option for patients with EBV-related NPC, our practice has changed. We have transitioned to using the GC regimen as an IC regimen.

The present study aims to retrospectively compare two IC regimens (GC versus DP) for patients with locally advanced NPC.

## Materials and methods

### Study design and participants

This is a retrospective study involving 105 cases of locally advanced NPC patients defined as stage III-IVA (American Joint Committee on Cancer (AJCC) between January 2016 and October 2022 at the Oncology Hospital ‘CHU Hassan II’ in Fez, Morocco. The inclusion criteria consisted of newly diagnosed, biopsy-proven non-metastatic NPC (8e AJCC), aged over 18, Karnofsky performance score (KPS) >70%, first-line IC for at least 2 cycles followed by CCRT and adequate renal function. Patients in the GC group received IC comprising gemcitabine (1,000 mg per square meter, days 1 and 8, intravenous infusion) and cisplatin (80 mg per square meter of body surface area, day 1, intravenous infusion for 3 cycles). Patients in the DP group received chemotherapy consisting of doxorubicin (60 mg per square meter, day 1, intravenous infusion) plus cisplatin (80 mg per square meter, day 1, intravenous infusion) every 3 weeks for 3 cycles. The concurrent chemotherapy regimen consisted of cisplatin (100 mg per square meter, intravenous infusion) every 3 weeks for 2–3 cycles during radiotherapy. Routine staging before treatment included a detailed medical history and a complete physical examination, nasopharyngoscopy with fiber-optic biopsy, MRI of the head and neck region, chest CT scan, abdominal ultrasound or CT scan, whole-body bone scintigraphy, as well as a complete blood count, comprehensive serum biochemical profile and ECG; EBV analysis was not done. All patients were restaged according to the 2010 AJCC staging system. CT scans of the head and neck, were performed and at the end of the IC, or in the event of clinical disease progression. The radiological assessment has been centrally reviewed by the same radiologist for all study participants and categorised using the RECIST criteria. Toxicity has been assessed before each cycle including haematological tests.

### Statistical analysis

Patient characteristics were compared using the chi-square test or Fisher's exact test. Statistical analyses were performed using SPSS software v26, and progression-free survival (PFS) and overall survival (OS) analyses were conducted using the Kaplan-Meier method with log-rank tests to assess differences between groups.

Treatment-induced toxicities were graded and classified according to the National Cancer Institute Common Terminology Criteria for Adverse Events, version 3.0.

## Results

A total of 105 cases were included. Table 1 provides an overview of the general clinical characteristics of the two groups. Across the entire cohort, the rates of complete response (CR), partial response (PR), stable disease (SD) and progressive disease (PD) were 3% (*n* = 3), 51% (*n* = 54), 31% (*n* = 33) and 15% (*n* = 15), respectively. The objective response rate (ORR) was 54% (*n* = 57). Within the DP group, the rates of CR, PR, SD and PD were 3.0% (*n* = 2), 60% (*n* = 37), 26% (*n* = 16) and 11% (*n* = 7), respectively. In the GP group, the incidence rates of CR, PR, SD and PD were 2% (*n* = 1), 39.5% (*n* = 17), 39.5% (*n* = 17) and 19% (*n* = 8), respectively. There were significant differences in PR rates (51% versus 39.5%, *p* = 0.028) between the DP and GP groups, favouring the DP regimen.

**Figure 1.**
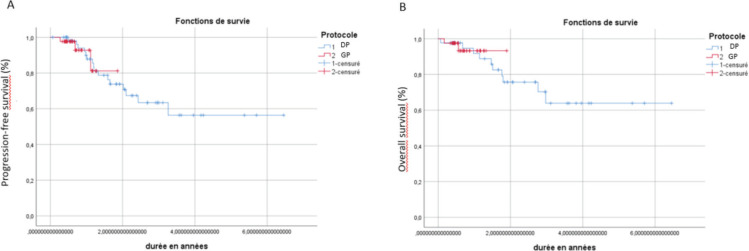
Kaplan–Meier curves of PFS (a) and OS (b) for patients with locally advanced NPC receiving DP or GP induction regimens.

The median follow-up duration was 27 months (5.3–82). The 2-year disease-free survival (DFS) was 70% in the DP IC group compared to 80% in the GP treatment group (*p* = 0.36) (Figure 1). The 2-year OS was 75% in the DP group and 90% in the GP group (*p* = 0.48) and no statistically significant survival difference was found between the two groups (Figure 1).

Patients in the DP group exhibited less grade 3–4 thrombocytopenia (*p* > 0.05) but more grade 3–4 leukopenia and neutropenia compared with those in the GP group (*p* > 0.05).

## Discussion

Concurrent platinum-based chemoradiotherapy constitutes the standard of care for patients with advanced NPC [8–10]. However, nearly 30% of these patients may experience local recurrence or distant metastases and treatment failure [8, 9].

As a result, several trials have evaluated the role of IC followed by CCRT. A meta-analysis that performed an individual patient data analysis of 1,193 patients included in 4 randomised trials, with a median follow-up of 5 years, concluded that the addition of IC to CCRT significantly prolonged both PFS (HR = 0.70, 95% confidence interval, 0.56–0.86 *p* = 0.0009) and OS (HR = 0.75, 95% CI 0.57–0.99, *p* = 0.04). This led to a 5-year absolute benefit of 9.3% and 5.5%, respectively. IC+CCRT also reduced rates of distant failure (HR = 0.68, 95% CI 0.51–0.90, *p* = 0.008) and had a tendency to improve local control (HR = 0.70, 95% CI 0.48 to 1.01, *p* = 0.06) [7]. Interestingly, no differences in survival were observed among different IC regimens, including TPF, gemcitabine-carboplatin-paclitaxel, TP (docetaxel-cisplatin), PF (cisplatin- 5FU) were detected. However, it is worth noting that no trial with anthracycline-based regimen has been included in this meta-analysis, nor did it include a GP regimen [11].

The GP regimen has been widely used and studied in recurrent or metastatic NPC and may extend PFS with acceptable adverse events [12]. Recent studies have shown that GP therapy administered in the induction phase achieves favourable clinical outcomes without severe toxicities [13]. In a Chinese phase III trial published in the New England Journal of Medicine, Zhang *et al* [13] retrospectively reported that the GP regimen had a positive effect on OS and showed a trend toward better DFS. The GP regimen could potentially be superior to the TP and PF regimens in the treatment of locally advanced NPC. However, the number of patients receiving GP regimen NACT in this study was limited, with only 13 patients in the GP group.

The combination of anthracycline and cisplatin has a lower level of evidence. The combination of epirubicin and cisplatin (EP) as IC was studied in a phase III trial by the Asian-Oceanian Clinical Oncology Association in patients with locally advanced undifferentiated or poorly differentiated NPC. The analysis showed no benefit from adding EP neoadjuvant chemotherapy to CCRT for patients with locally advanced NPC, although the overall incidence of recurrence was reduced with the addition of EP to CCRT,

In Morocco, historically, the IC regimens for the treatment of locally advanced NPC mainly consisted of PF or DP. Currently, the recommended regimen is GP based on the Asiatic phase III trial conducted by Zhang *et al* [13].

Given the absence of a confirmatory trial conducted in Morocco, we decided to evaluate the efficacy and safety data of a patient cohort treated with the newly adopted GP regimen and compare it to a historical cohort of patients treated with DP.

In our study, the overall response rate to IC chemotherapy was lower than that reported in the literature, and particularly compared to the findings by Zhang *et al* [13] with the GP induction regimen (54% versus 94.5%, respectively). The lower response rates observed in our study should be confirmed by a larger prospective trial, which could include tumour biomarkers and investigate pharmacogenetic factors to explore the reasons behind this possible reduced chemosensitivity. It has been demonstrated that the EBV DNA can predict the disease prognosis and even guide chemotherapy. It should be adopted to complement the TNM classification in selecting high-risk patients with NPC. Our study did not assess the relationship between EBV status and chemotherapy sensitivity.

However, we notably found that patients receiving DP-based induction achieved a significantly higher response rate than those receiving the GP regimen with 60% achieving PR compared to 39.5% in the GP regimen.

Furthermore, we observed lower PFS and OS rates at 2 years, which could be attributed to either more biologically aggressive disease or reduced chemosensitivity.

This study has a lot of limitations, including itsretrospective nature, the small number of patients and a comparison to a historical cohort. However, the significance of these findings should not be overlooked. When considering the final report of Zhang *et al* [13] in which they demonstrated a significant positive correlation between the depth of tumour response to IC (CR versus PR versus SD/PD) and survival (5-year OS of 100% versus 88.4% versus 61.5% *p* = 0.005), and despite the absence of statistically significant differences in 2 years in PFS or OS between DP and GP, these findings raise questions about adopting GP for our patients.

We only reported the 2-year survival outcomes and acute toxicities in this study, and assessing OS and long-term adverse events related to treatment may require a longer follow-up period. We believe that a confirmatory randomised trial is warranted to investigate the optimal IC in locally advanced NPC in the Moroccan population.

## Conclusion

In summary, our study concluded that a DP induction regimen was superior to GP regimens for locally advanced high-risk NPC in terms of response rate with acceptable toxicities. Further studies are needed to validate our results and to inform the local guidelines.

## Conflicts of interest

No conflicts of interest.

## Funding

Our research project was not sponsored.

## Figures and Tables

**Table 1.** Patient characteristics.

Characteristic	DP group (*N* = 62)	GP group (*N* = 43)	*p*-value
Median age years (19–79)	51 (20–77)	47 (19–79)	0.77
Sex – no. (%)			0.8
Male	34 (54.8%)	30(69,7%)	
Female	28 (45%)	13(30%)	
Karnofsky performance – status score			0.65
100	13 (20.9%)	10 (23.25%)	
90	45 (72.5%)	20 (46.5%)	
80	4 (6.45%)	10 (23.25%)	
70	0	3 (6.97%)	
Histology			0.89
Differentiated	3 (4.83%)	2 (4.65%)	
Undifferentiated	59 (95%)	41 (95.34%)	
Tumor category – no. (%)			0.86
T1	2 (3.22%)	4 (9.3%)	
T2	12 (19.35%)	11 (23.58%)	
T3	21 (33.8%)	10 (23.25%)	
T4	27 (43.5%)	18 (41.86%)	
Node category – no. (%)			0.76
N0	6 (9.67%)	6 (13.95%)	
N1	18 (29%)	12 (27.9%)	
N2	25 (40.3%)	20 (46.5%)	
N3A	9 (14.5%)	3 (6.97%)	
N3B	4 (6.45%)	2 (4.65%)	
Disease stage – no. (%)			0.91
III	40 (64.5%)	30 (69.76%)	
IVA	22 (35.48%)	13 (30.23%)	

## References

[1] Parkin DM, Bray F, Ferlay J (2005). Statistiques mondiales sur le cancer, 2002. CA Cancer J Clin.

[2] Ferlay J, Ervik M, Lam F (2018). Global Cancer Observatory: Cancer Today.

[3] OuYang PY, Su Z, Ma XH (2013). Comparison of TNM staging systems for nasopharyngeal carcinoma and proposal of a new staging system. Br J Cancer.

[4] Alami Z, Bouhafa T, Elmazghi A (2018). The contribution of concomitant radiochemotherapy in the management of undifferentiated nasopharyngeal carcinoma in adults. Pan Afr Med J.

[5] Abdellatif B, Karima B (2016). The cancer registry of the greater Casablanca region for the period 2008–2012.

[6] Erratum world cancer statistics 2018: GLOBOCAN estimates of incidence and mortality worldwide for 36 cancers in 185 countries. CA Cancer J Clin.

[7] Wang P, Zhang M, Ke C (2019). The effectiveness and lightness of induction chemotherapy associated with concomitant chemoradiotherapy in locoregionally advanced nasopharyngeal carcinoma. Eur J Cancer.

[8] Lee AW, Ma BB, Ng WT (2015). Management of nasopharyngeal carcinoma: current practice and future perspective. J Clin Oncol.

[9] Lin JC, Jan JS, Hsu CY (2003). Phase III study comparing concurrent chemoradiotherapy with radiotherapy alone in advanced nasopharyngeal carcinoma: positive impact on overall and progression-free survival. J Clin Oncol.

[10] Al-Sarraf M, LeBlanc M, Giri PG (1998). Chemoradiotherapy vs. radiotherapy in patients with advanced nasopharyngeal carcinoma: phase III randomized intergroup study 0099. J Clin Oncol.

[11] Chen YP, Tang LL (2018). Induction chemotherapy plus concomitant chemoradiotherapy in endemic nasopharyngeal carcinoma. Clin Cancer Res.

[12] Ngan RKC, Yiu HHY, Lau WH (2002). Gemcitabine and cisplatin combination chemotherapy for metastatic or recurrent nasopharyngeal carcinoma. Ann Oncol Août.

[13] Zhang Y, Chen L, Hu GQ (2019). Chimiothérapie d'induction à la gemcitabine et au cisplatine dans le carcinome nasopharyngé. N Engl J Med.

